# Microstructured and Degradable Bacterial Cellulose–Gelatin Composite Membranes: Mineralization Aspects and Biomedical Relevance

**DOI:** 10.3390/nano9020303

**Published:** 2019-02-22

**Authors:** Selestina Gorgieva, Silvo Hribernik

**Affiliations:** Faculty of Mechanical Engineering, University of Maribor, 2000 Maribor, Slovenia; silvo.hribernik@um.si

**Keywords:** bacterial cellulose, gelatin, microstructuring, mineralization, guided tissue regeneration

## Abstract

Bacterial cellulose (BC)–gelatin (GEL) membranes were processed by successive periodate oxidation and a freeze-thawing/carbodiimide crosslinking procedure, first facilitating a Schiff-base reaction among respective aldehyde and hydroxyl groups, and later GEL stabilization and microstructuring. The formation of highly microporous structures within the GEL portion, with significant differences between bottom and top, was elucidated, and pores in the 27.6 ± 3 µm–108 ± 5 µm range were generated, exceeding the threshold value of ~10 µm sufficient for cell trafficking. During a relatively short (6 h) exhaustion procedure in supersaturated simulated body fluid solution, the membranes accommodated the combination of biologically relevant minerals, i.e., flake-like octacalcium phosphate (OCP) and (amorphous) apatite, onto their surface, forming a membrane with intensive swelling (650–1650%) and up to 90% weight loss in a 4-week period. The membranes´ 6-day eluates did not evoke any cytotoxic effects toward human fibroblast, MRC-5 cells. The same type of cells retained their morphology in direct contact with the membrane, attaching to the GEL porous site, while not attaching to the GEL thin-coated BC side, most probably due to combined, ablation effect of dominant β-sheet conformation and carbodiimide crosslinking. Together with arrested proliferation through the BC side, the membranes demonstrated beneficial properties for potential guided tissue regeneration (GTR) applications.

## 1. Introduction

Bacterial cellulose (BC) is a nanofibrilar, firmly structured polysaccharide, produced extracellularly by the gram-negative, acetic acid bacteria *Komagataeibacter xylinus* (the previous *Gluconcetobacter xylinus* [1]). This nanomaterial, consisting of randomly assembled, <100-nm-wide ribbon-shaped fibrils, is composed of 7–8-nm-wide elementary nanofibrils aggregated in bundles. It delivers a combination of exclusive properties, such as a high crystallinity (84–89%) and polymerization degree, high surface area (high aspect ratio of fibers with diameter 20–100 nm), elevated purity with the absence of lignin and hemicellulose, high flexibility and tensile strength (Young modulus of 15–18 GPa), high water-holding capacity (over 100 times of its own weight), etc. BC is a highly pure, noncytotoxic, nongenotoxic, and highly biocompatible material. This is primarily related to its chemical composition, as already demonstrated for cellulosic derivatives (e.g., cellulose acetate [2,3,4]), mainly to its biosafe degradation profile. As such, it attracts interest in diverse areas, with hallmarks in medicine, with a 30-year research history in the field. BC attracts interest for applications such as in the engineering of artificial skin (particularly in the recuperation of burned skin), artificial blood vessels, topical wound coverings, nerve surgery, dura mater prostheses, arterial stent coatings, wound dressings [5], hemostatic material, electronic platforms, implants for cartilage and bone repair, etc. The capacity to promote long-term stemness of mouse embryonic stem cells [6], as well as a supersoft neural interfacing substrate [7], have been proposed. Already commercialized BC products are used in the treatment of burns and wounds (Bioprocess^®^, BASYC^®^, Biofill^®^), in the treatment of periodontal defects (Gengiflex^®^), etc.

Composites containing BC outperform single BC on many terms, such as a higher water retention for the chitosan/BC composite [8], improved mechanical performance for polyvinyl alcohol (PVA)/BC composites [9], cell ingrowth promotion in polyethylene glycol (PEG)/BC composites, blood clot control in kaolin/BC composites [10], hemocompatibility in peptide (Arg-Gly-Asp)/BC materials [11], etc. The frequently exposed problem of BC itself regarding its medical applicability (i.e., regenerative medicine and tissue engineering) is a lack of bioactivity, translated as a lack of cell recognition moieties, intrinsic antimicrobial activity, mineralization centers, etc. Diverse and vast numbers of in situ and ex situ modification procedures have been investigated to cope with those issues, as a way toward bionic design, with an emphasis on extracellular matrix (ECM) recapitulation [12]. The straightforward approach is the addition of reinforcement material (chitosan, gelatin, poly-3-hydroxybutirate, nanomaterials, clays, silica) to the bacterial culture medium, which integrates well inside the growing BC fibrils. However, the effectiveness of such an approach has often been hindered by toxicity toward BC-producing bacteria, the insolubility of various materials in culture media, particle size limitation for effective penetration within BC, high surface tension toward hydrophobic materials, etc. The homogenization or dissolving of BC and further mixing with a material of choice [13], or postsynthetic periodate oxidation and grafting [14], are other approaches to cope with the obstacles of in situ modification methods. BC compounding with bioactive materials (proteins, inorganic calcium phosphates, etc.) is another efficient modification model, applicable in biomedical research in the tracking of tumor cell behavior [15], osteoblast cell growth in bone regeneration [16,17], fibroblast/endothelial cell guides in wound healing, etc. In most cases, the compounding polymers (or proteins) are applied in the form of a thin coating around the BC, rather than a sterically structured layer, resembling the native ECM. There have been a limited number of studies dealing with structuring phenomena within BC-containing composites, as well as some that have featured phenomena (in situ mineralization, degradation) related to particular biomedical applications.

Herein, we developed composite membranes, combining BC membranes and gelatin (GEL) biopolymers, and further evaluated their feasibility as a biomedical material. Several aspects were investigated and discussed (conjugation, microstructuring, in situ mineralization outcomes, and biodegradation) in relation to processing conditions as well as further biological applications. We hypothesized the following:

1. A BC membrane can be efficiently merged with porous GEL through a simple procedure we propose, using a water-based system free of including additional chemicals;
2. Short incubation in supersaturated simulated body fluid (SBF) promotes the formation of calcium phosphate (CaP)-based minerals on and within BC–GEL composites; and
3. Human embryo fibroblast cells attaches differently on differently structured GELs.

Both materials used were assumed to be biomimetic, the BC in terms of (nanofibrilar) morphology and GEL as a chemical counterpart of collagen, and therefore developing mimetic (extracellular-like) microstructures would add another mimetic dimension onto the developed material. Moreover, short-term mineralization (also relying on exhaustion as a biomimetic concept) is often proposed for metallic implants, but is rarely seen and elaborated in ˝soft˝ biopolymer-based materials. Finally, a biological assessment involving human embryo fibroblast in direct contact and in an indirect (elution) test with developed membranes was intended to identify and suggest composite membranes’ applications in guided tissue regeneration procedures.

## 2. Materials and Methods

### 2.1. Reagents and Materials

Purified and autoclaved BC membranes were obtained from Fzmb (GmbH Research Centre of Medical Technology and Biotechnology, Bad Langensalza, Germany). GEL from bovine skin (Mw of 15–80 kDa [18]), and fluorescein isothiocyanate (FITC), CH_3_COOH, NaH_2_PO_4_, KCl, NaCl, CaCl_2_·2H_2_O, MgCl_2_·6H_2_O, *N*-hydroxysuccinimide (NHS), 1-ethyl-3(3-dimethylaminopropyl)-1-carbodiimide hydrochloride (EDC), and 2-(N-morpholino)ethanesulfonic acid (MES) were purchased from Sigma Aldrich (Taufkirchen, Germany). Minimal Essential Medium (MEM) was obtained from Life technologies (Carlsbad, CA, USA), and fetal bovine serum was obtained from Gibco (Dublin, Ireland). All chemicals were analytical grade.

### 2.2. BC–GEL Membranes

BC membranes with dimensions of 400 × 400 × 2 µm were immersed in 10 mL of 1% or 2% sodium periodate (NaIO_4_) and further stirred (at 70 rpm) for 24 h in the dark. Afterward, membranes were thoroughly rinsed, first with ethylene glycol to remove remaining sodium periodate and after with distilled water. Native and periodate-treated BC was positioned on the bottom of a glass Petri dish (with *d* = 45 mm) having an antistick Teflon liner. GEL solutions (5% and 10% w/v) were prepared in parallel by dissolving suitable amounts of GEL in 0.1 M MES buffer and adjusting the pH to 5.5. In addition, 2 mL of GEL solution were mixed with 150 µL EDC and NHS water solutions, containing 16.3 mg EDC/19.6 mg NHS for 5% GEL and 32.6 mg EDC/39.2 mg NHS for 10% GEL. GEL solutions were immediately poured on top of the BC membranes. Glass Petri dishes were put in a freezer at −20 °C for 4 h and afterward removed and left to thaw in a cooler at 8 °C for an additional 1 day. The excess of EDC and NHS and reaction side-product (urea derivative) was removed by washing with a 70/30 EtOH/Milly Q water solution, which was expected to disinfect the membrane to a certain extent. Membranes were kept in a cold and dry place with the following characterizations.

### 2.3. In Situ Mineralization

For deposition of calcium phosphate-based minerals, a relatively fast biomimetic procedure was used [19]. A 10× SBF solution was prepared by successive dissolution of the following amounts of respective salts: 116.8860 g NaCl, 0.7456 g KCl, 7.3508 g CaCl_2_·2H_2_O, 2.0330 g MgCl_2_·6H_2_O, and 2.3996 g NaH_2_PO_4_ in 2 L of Milli-Q water. The resulting solution had a pH of 4.4. Afterwards, the membrane sample was immersed in 200 mL of prepared solution, and NaHCO_3_ (10 mM) was further dissolved in it. Membranes were kept immersed with slow shaking for 6 h, subsequently washed with Milli-Q water, frozen, and lyophilized.

### 2.4. Fourier Transform Infrared (FTIR) Spectroscopy

A Perkin–Elmer IR spectrophotometer (Waltham, MA, USA) with an Attenuated Total Reflectance (ATR) attachment was used in the analysis within the 4000–450 cm^−1^ range. Sixteen scans were accumulated for each sample, utilizing a resolution of 4 cm^−1^. For data analysis, Spectrum 5.0.2 software (Waltham, MA, USA) was applied. In order to enhance the resolution of overlapping peaks in regions of interest, inspection of second derivatives on spectra was done using a Savizky–Golay algorithm with 3 smoothing points.

### 2.5. X-Ray Diffraction (XRD) Spectroscopy

The crystallography pattern of composite membranes was obtained using a diffractometer D4 Endeavor (Bruker, Billerica, MA, USA) with Cu Kα radiation (λ = 1.5406 A) in a continuous scan mode and an energy dispersive detector from Sol-XE (Bruker, Karlsruhe, Germany). A filament current of 30 mA and an acceleration voltage of 45 kV were applied. Diffraction angles were between 5° and 80°, while the scan step was 0.02°. By using JCPDS standard cards, the type of formed minerals was evaluated.

### 2.6. Scanning Electron Microscopy (SEM) and Energy Dispersive X-Ray (EDX) Spectroscopy

The morphology of (oxidized and GEL-coupled) BC membranes was evaluated by means of SEM imaging on a microscope Supra 35 VP (Carl Zeiss, Yena, Germany) using secondary electron modes and up to 150,000× magnification. SEM coupled with an EDX detection system (detector Inca 400, Oxford Instruments, Paris, France) was further used for mineral evaluation, atomic inspection, and elemental identification of deposited minerals. Samples were, prior to imaging and EDX analysis, sputtered with a thin layer of palladium.

### 2.7. Confocal Fluorescence Microscopy (CFM)

The microstructure of composite membranes (their top, bottom, and cross-section, respective to freezing plate position) in a wet state and in confocal mode was assessed by confocal fluorescence microscopy (CFM) using an inverted CFM Leica TCS SP5 II (Wetzlar, Germany) equipped with an LAS AF software program (Wetzlar, Germany). Membranes were positioned on a glass holder above the dry objective (×20). FITC-labeled GEL was detected using the color coding function, and FITC excitation was done with an argon laser. Light was collected on a hybrid HyD3 detector using the FITC probe settings, with an excitation of 492 nm and an emission of 512 nm. In addition, 1024 × 1024 pixel images were obtained by light gain correction and an 8× line averaging function. Several places of membrane were analyzed. Fluorescence and bright field channels were overlaid, and images were analyzed by the ImageJ program plug-in BoneJ (http://imagej.nih.gov/ij/). The preparation of images and data analysis is further described in “Results and Discussion”.

### 2.8. Swelling and Degradation Test

Membranes with different compositions were immersed in 100 mL of PBS. At predetermined periods (up to 90 min), the wet membranes were weighed, and the swelling percentage was calculated using the normalized dry-weight difference between wet and dry membranes multiplied by 100. The degradation of membranes was evaluated by immersing round pieces of membranes with *d* = 15 mm in 20 mL of SBF [20] at 37 °C. After 1–4 weeks incubation, immersed pieces were taken out, washed, dried, and a percentage of weight loss was calculated. Simultaneously, the pH media after membrane degradation were measured.

### 2.9. Cell Testing

#### 2.9.1. Cell Line

MRC-5 cells (ATCC^®^ CCL-171™, Wesel, Germany) were derived from the normal lung tissues of a 14-week-old male fetus. They were primarily cultured in Costar Corning culture flasks in MEM medium, with an addition of 10% fetal bovine serum, 2 mM L-glutamine, Earle’s salts, 1% penicillin/streptomycin (Sigma Aldrich, Taufkirchen, Germany) at 37 °C, and 5% CO_2_.

#### 2.9.2. Preparation of Eluates and Cellular Exposure

Samples were delivered in dry form and were hydrated for one day in 70% ethanol with three changes. Afterwards, samples were rinsed 10 times during 2 days with water to remove the ethanol. Samples (3 cm^2^ from the central square part) were extracted in cell culture medium for 24 h at 37 °C according to ISO10993-1 guidelines. Only the dense sides of the samples were used for the preparation. The pH of the eluate was between 6 and 7. To obtain subconfluent cultures, 11,000 MRC-5 cells were seeded per well and cultured 24 h prior to the exposure with the samples. Eluates were applied in the following concentrations: Pure, 1 + 1, 1 + 4, 1 + 9, and 1 + 19. Cells were cultured for 72 h. The positive control was the addition of 2 μL of lysis reagent (70% EtOH + TritonX 100 (Sigma Aldrich, Taufkirchen, Germany)) for 10 min. Cell culture medium was used as a negative control.

#### 2.9.3. Direct Exposure of Cells with Samples

MRC-5 cells were seeded directly onto the membranes’ upper side. Control cells were seeded on plastic wells. Exposures were checked under the microscope. The area around the samples was also checked, since seeded cells usually do not grow exclusively on the samples but also adhere to the surrounding well. A visual check for changes in morphology and cell density was performed by microscopy after 4, 24, 48, 72, and 96 h (the last time point was only for testing in direct contact).

#### 2.9.4. Bioreduction into Formazan

For testing, a CellTiter 96^®^ Aqueous Non-Radioactive Cell Proliferation Assay (Promega, Madison, WI, USA) was used. MTS solution and PMS solution (Sigma Aldrich, Taufkirchen, Germany) were thawed, 100 μL of the PMS solution was mixed with 2 mL of the MTS solution, and 20 μL of the combined MTS/PMS solution was added to 100 μL of each well. Plates were incubated in a cell incubator for 2 h at 37 °C and 5% CO_2_. Absorbance at 490 nm was measured on a plate reader (SPECTRA MAX plus 384, Molecular Devices, San Jose, CA, USA). Dehydrogenase activity, which correlates with cell viability, was calculated from Equation (1):

(1) $$Dehydrogenase\;Activity\;\left(\%\right)=100\times \frac{\left(A_{450\mathrm{nm}\;\mathrm{sample}}-A_{450\;\mathrm{nm}\;\mathrm{control}}\right)}{\left(A_{490\mathrm{nm}\;\mathrm{sample}}-A_{490\;\mathrm{nm}\;\mathrm{control}}\right)}$$

#### 2.9.5. Analysis in Direct Contact

MRC-5 cells (60,000 cells) were seeded on top of the membranes and cultured for 96 h. After staining with Hoechst 44032 (1 μg/mL) for 15 min at 37 °C, the samples were triple-washed with PBS, and the samples were stored in liquid nitrogen. Sections (20 μm thick) were cut on a cryostat microtome HM 560M and viewed using an Olympus BX 51 microscope (Vienna, Austria). Due to the autofluorescence of the samples, it was not easy to discriminate the nuclear stain from the sample. A combination of a phase contrast image with fluorescence in the blue channel worked suboptimally. Based on the spectrum of the samples, autofluorescence images were taken in different fluorescence channels to discriminate the cell staining. Nuclear staining with Hoechst 33342 was imaged using settings for blue dyes (λ_ex_ 330–385 nm, λ_em_ 420 nm). Autofluorescence was detected using settings for green dyes (λ_ex_ 460–490 nm, λ_em_ 510–550 nm). At these settings, staining with Hoechst 33342 was not seen. Merging of the images obtained by the different settings could be used to discriminate the nuclear signal from the autofluorescence. Samples without sectioning (whole mounts) were viewed on an LSM 510 Meta Laser Scan microscope (Jena, Germany) with λ_ex_ 405 nm and a bandpass 420–480-nm filter.

#### 2.9.6. Statistical Analysis

Data were reported as mean ± standard error. One-way ANOVA or two-way ANOVA were used for data analysis using IBM SPSS Statistics software (New York, NY, USA). Statistical significance was accepted at *p* ≤ 0.05.

## 3. Results and Discussion

### 3.1. BC Modification: Oxidation and Conjugation with GEL

**Figure 1:**
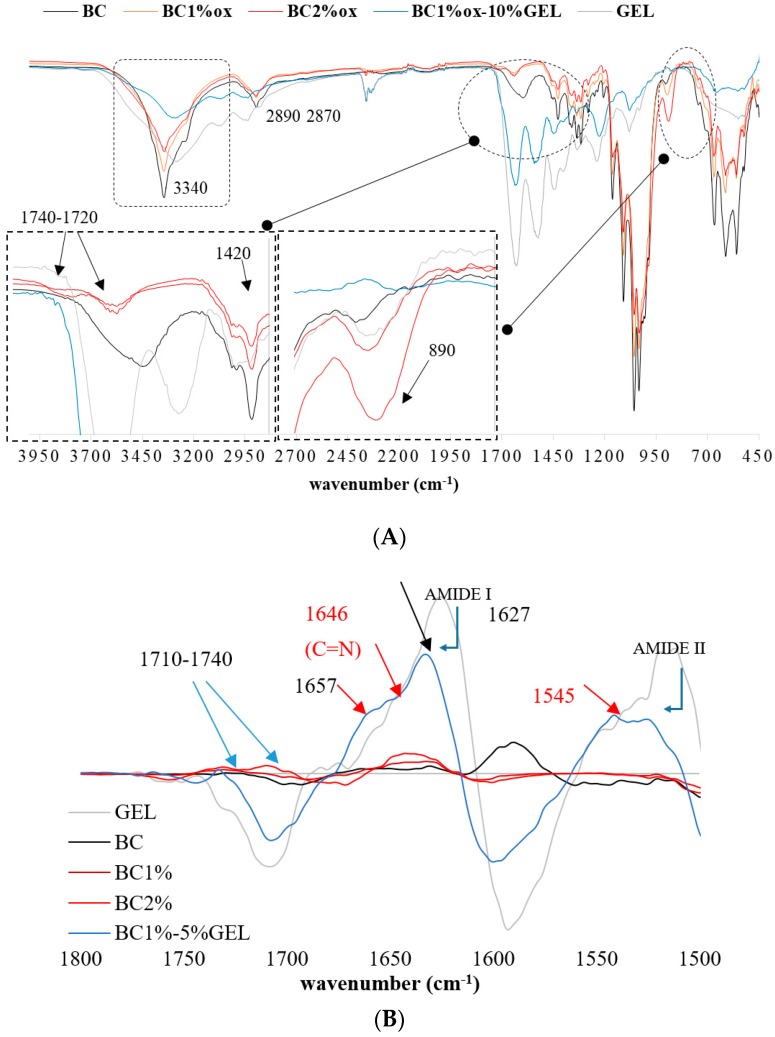
(**A**) Fourier Transform Infrared (FTIR) spectra of native, 1%, and 2% periodate-oxidized bacterial cellulose (BC) and BC1%ox–10%GEL (gelatin) and (**B**) FTIR spectra of the second derivative for the region between 1500 and 1800 cm^−1^.

The periodate-mediated oxidation of BC is reported to proceed through redox cleavage of vicinal (C2−C3) glycols, which yields a product with aldehyde groups in the positions C2 and C3 of the glucopiranose unit, i.e., 2,3-dialdehyde BC [21]. The concentration of periodate is a major factor affecting the extent of region-selective oxidation, and therefore we screened several concentrations and selected 1% and 2% based on the preserved integrity of BC. The inspection of the FTIR spectra (Figure 1a) of native BC confirmed the typical bands for BC, assigned as follows: A broad signal in the 3200–3600 cm^−1^ region related to an intramolecular hydrogen bond for O3…H–O5 [22], its center at 3340 cm^−1^ related to an OH stretching vibration, 2870 cm^−1^ and 2890 cm^−1^ related to C–H stretching in CH and CH_2_, 1355 cm^−1^ related to symmetric bending of CH_2_, and bands at 1170–1000 cm^−1^ related to O–H, C–H, and C–O–C bending vibrations. Compared to the native, the periodate-treated BC exhibited new bands, first in the 1720–1740 cm^−1^ region, and next at 890 cm^−1^, related to the aldehyde group and its hemiacetal or hydrated form, respectively [23]. Indeed, periodate oxidation may involve the formation of different structures, i.e., free or hydrated aldehydes, hemialdols, and hemiacetals [24] with simultaneous hydrolytic cleavage of glycoside bonds affecting BC crystallinity. Herein, the total crystalline index (TCI) was calculated from the *A* 1370 cm^−1^/*A* 2900 cm^–1^ ration, which was 0.83 for nontreated BC, while 1% and 2% periodate oxidation reduced it to 0.64 and 0.54, respectively. Other indicators of crystallinity change were bands at ~1420 and ~890 cm^–1^ (Figure 1a, inserts), which were directly related to crystalline and amorphous structures, respectively, again demonstrating an obvious increase in the amorphous part at the expense of crystallinity after periodate oxidation.

A typical spectroscopic signature of GEL was also evidenced by FTIR, i.e., absorption at 3410 cm^−1^ assigned to N–H stretching, 1640 cm^−1^ to C=O stretching, 1550 cm^−1^ to C–N stretching, and 1247 cm^−1^ to N–H bending. Obviously, the GEL component dominated in the BC–GEL composite due to mass prevalence, as well as complete BC entrapment within the membrane. According to the literature [25], the aldehyde groups from oxidized BC may further cross-link with lysine or hydroxylysine, ε-amino groups of GEL by Schiff’s base. This phenomenon was hardly visible in our situation due to large peak overlapping.

To enhance the separation and peak resolution in the 1500–1800 cm^−1^ region, the second derivative spectra were inspected (Figure 1B) and, due to the transmission mode, the local maximums within the deconvoluted spectra were inserted. A new vibration at 1646 cm^−1^ was observed, as well as a change in the Amide II region, both being related to Schiff-base formation [20,26]. Very similar FTIR data were obtained for all membranes (data not presented). Reduction and small red shifts of Amide I and II peaks in BC–GEL relative to GEL was due to EDC cross-linking, which generated new amide bonds by conjugating free COOH and NH_2_ groups from GEL-amino acids, i.e., glutamic/aspartic acid and lysine/hydroxylysine side chains, respectively. Such conjugation enhanced the physiological stability of GEL far above its sol-gel transition temperature (~30 °C) [27]. The second derivative data from the Amide I band region disclosed other important information, applicable in the biological evaluation of the membrane. Indeed, a prominent peak at ~1650 cm^−1^, arising from a C=O stretching vibration of the protein backbone, was essentially sensitive to secondary structures and different hydrogen-bonding environments for protein conformations α-helix, β-sheet, turns, and unordered types [28]. In the analyzed membranes, a dominant β-sheet structure was observed at 1627 cm^−1^, with the minor contribution of an amorphous random coil (at >1646 cm^−1^) and an ordered helix (at >1655 cm^−1^) [29]. We believe that such conformation resulted from GEL gelling at confined conditions, meaning within the structure already arrested by EDC cross-linking, very similarly to the effect observed recently for gelatin microgels [30]. This is important from the point of cell adhesion to such materials, since the cell recognition sequence within the GEL (the linear RGD motif) was expected to be less exposed to cell integrins when they were in an ordered instead of unordered conformation.

### 3.2. Microstructural Assessment

**Figure 2:**
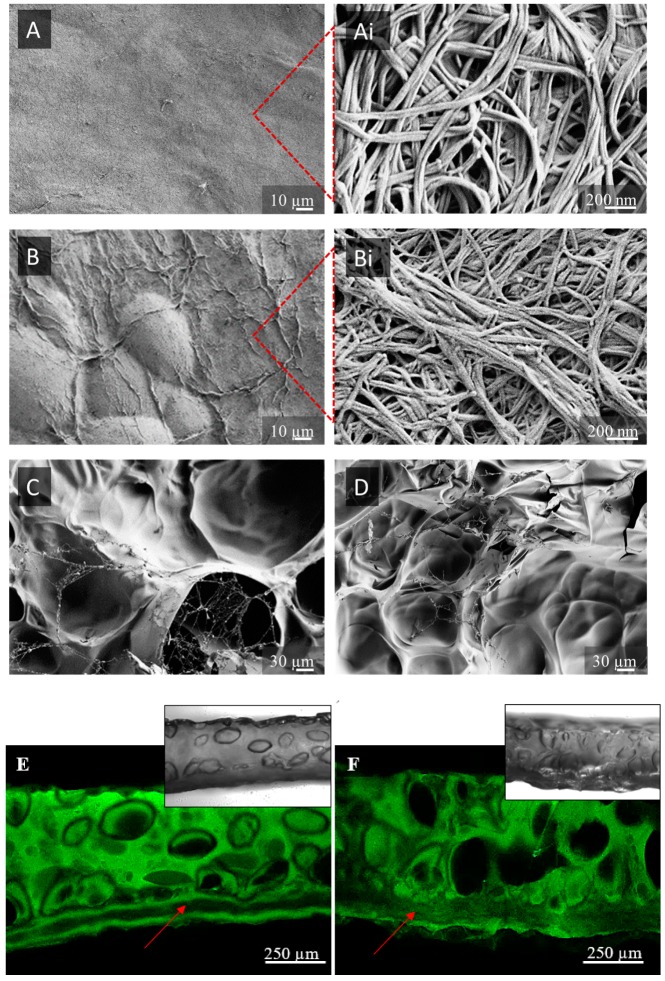
Scanning electron microscopy (SEM) micrographs of BC before (**A**,**Ai**) and after (**B**,**Bi**) oxidation with 2% sodium periodate; and coating with 5% GEL (**C**); and 10% GEL (**D**); and confocal fluorescence microscopy (CFM) images from top cross-section of BC–GEL membranes containing nontreated (**E**); and 2% oxidized BC (**F**). Respective bright field micrographs were inserted into fluorescence (FITC)-aided images.

SEM micrographs presented in Figure 2 depict the top surface of membranes, demonstrating a typical BC network structure with unique micromorphology, visible at high magnification (Figure 2A,Ai). Isotopically organized nanofibers were not significantly compromised after the oxidation process, even at 2% periodate concentration (Figure 2B,Bi). Before oxidation, a 3D network of loosely arranged nanofibers with larger pores was visible. Concentration of periodate and treatment duration affected the surface of BC fibrils, evoking merging between neighboring nanofibrils, as observed within higher magnification inserts, which translated to a reduction in nanoporosity, compacting, and shrinking at the macroscale with close to twice the reduction of membrane dimensions in 2% periodate treatment and not significant thickness reduction [21]. As suggested by FTIR, this could have been a consequence of the Schiff-base reaction, as well as reduced crystallinity, allowing closer intermolecular contact and (H–) bonding between formed aldehydes and surrounding hydroxyls.

Due to the hydrophilicity of both components, as well as the high surface area of BC nanofibrils, the GEL coating continuously covered the BC surface (Figure 2C,D). The intensive integration between GEL and BC relied on electrostatic interactions. Indeed, due to the electron withdrawing nature of the carbonyl group within the GEL protein, the electron ion pair on the nitrogen was delocalized by resonance, forming a partial double bond with carbonyl carbon and a negative charge on oxygen on one side and a positive on the nitrogen atom on another [31]. The BC, rich with electronegative OH groups, adsorbed these cations, causing intensive adsorption onto the BC, which we assumed further supplemented the Schiff-base formation in oxidized BC by reducing the distance between involved functionalities.

The top-site open porosity of GEL was not prominent, which was an artifact of the freeze-drying process required for SEM imaging. Another issue was providing the representative cross-sections due to compressibility and bendability of the BC–GEL membranes in dry state. The CFM, a superior technique to SEM in wet-state imaging was used for top and bottom (respective to freezing plate) side (Figure S1) and imaging of cross-sections (Figure 2E,F). The latter micrographs demonstrate anisotropic structuring within two structurally distinct regions: dense BC from one side and porous GEL, on top of it “enveloping” the BC membrane. Close integration between distinct regions result in compact composite material, mainly due to high surface area of BC available for coating with GEL [32] and already described electrostatic interactions. Such structuring profile is expected to provide efficient barrier towards cell proliferation (dense part) and regenerative function (porous part) within intended guided tissue regeneration (GTR) application. Extensive GEL penetration within the membrane was visible within 2% oxidized BC (FITC signal within the BC section) due to the presence of an accessible, amorphous part within the BC bulk, mainly responsible for the enhanced degradation process (described within the physiological data section). As oxidation of BC occurs under heterogeneous conditions, it was expected that the surface of BC fibrils would be mainly affected, rather than their bulk, which allowed partial penetration of GEL, intensifying further their integration, yet keeping the BC membrane integrity. As BC was not fluorescently labeled, bright field images (inserts) were also obtained to demonstrate its presence (Figure 2E,F, bottom).

**Figure 3:**
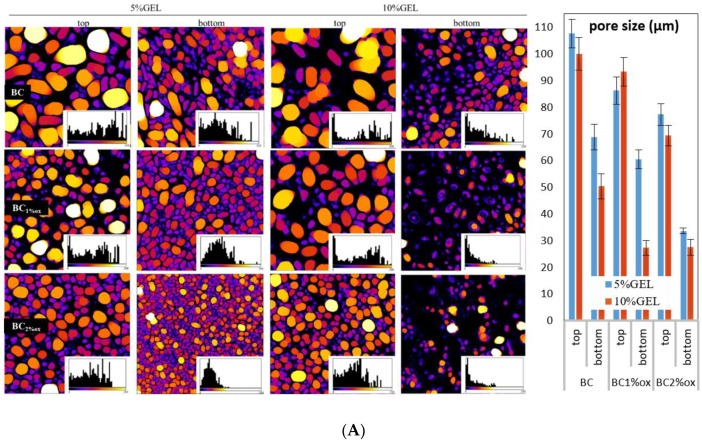

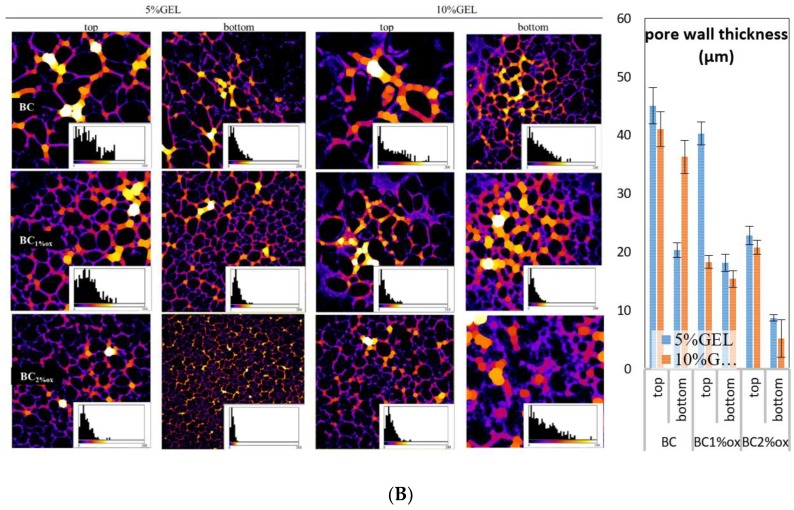
Color-coded images of BC–GEL membranes emphasizing pores (**A**) and pore walls (**B**). The image insets are respective histograms demonstrating normalized distributions of pore size and pore wall thicknesses within the tops and bottoms of membranes (*x* axis: 0–200 µm, *y* axis: count) without and with 5% and 10% GEL, and (non)oxidized BC. Mean/SD data of pore size and pore wall thickness are presented next to respective (**A**,**B**) image sets.

A quantitative data assessment of microstructure parameters from top and bottom micrographs was performed using the ImageJ program (BoneJ plugin). Originally, acquired CFM micrographs were pre-processed (noise, dark, with outlier elimination) and calculated by a thickness function resulting in color-coded maps and respective histograms (Figure 3). The distribution of diameters in pore spaces as well as in pore wall spaces was obtained, which correlated with pore size and wall thickness. The applied color-coding supplemented the visual perception of uniformity and distributions within a particular membrane.

Pore size data for hydrated membranes disclosed three general relations: BC > BC1%ox > BC2%ox, top > bottom, and 5% GEL > 10% GEL. The presence of a nanostructured BC membrane on a freeze-plate-contacting (bottom) part during the freezing process affected the size of pores [33] since it modulated the heat transfer through the membranes in a way that slowed down the freezing front progression from bottom to top when the underlying BC membrane was dense. This ensured sufficient time for nucleated ice crystals (the pore templates) to grow at a particular level. Moreover, the freezing process slowed down from bottom to top again, allowing pore templates to ˝grow˝ at membranes’ top proximity, thus making pores larger on top than the bottom. On the other hand, GEL concentration affected the size due to separation within the solution into polymer-rich (GEL) and lean phases (the water). Water dominated in 5% GEL, which again supported the ice crystals growing at the freezing stage, making a significant difference between pore size on bottom and top. Close relations between both pore size and wall thickness translated into very similar thickness data profiles. In summary, the BC structure affected by the periodate treatment significantly impacted the microstructure of the GEL segment, generating pores in the 27.6 ± 3 µm–108 ± 5 µm range, all exceeding the threshold value of ~10 µm sufficient for cell trafficking [34].

### 3.3. Mineralization

The mineralization experiment was intended to supplement the understanding of the actions of BC–GEL conjugates as biomolecular motifs for the formation of biologically relevant minerals. Precisely, the membranes were inspected in terms of whether they could support the precipitation of CaP-based minerals (and which types) within relatively short (6 h) incubations into supersaturated saline solution, advantageous over classical mineralization procedures in terms of energy and time effort.

**Figure 4:**
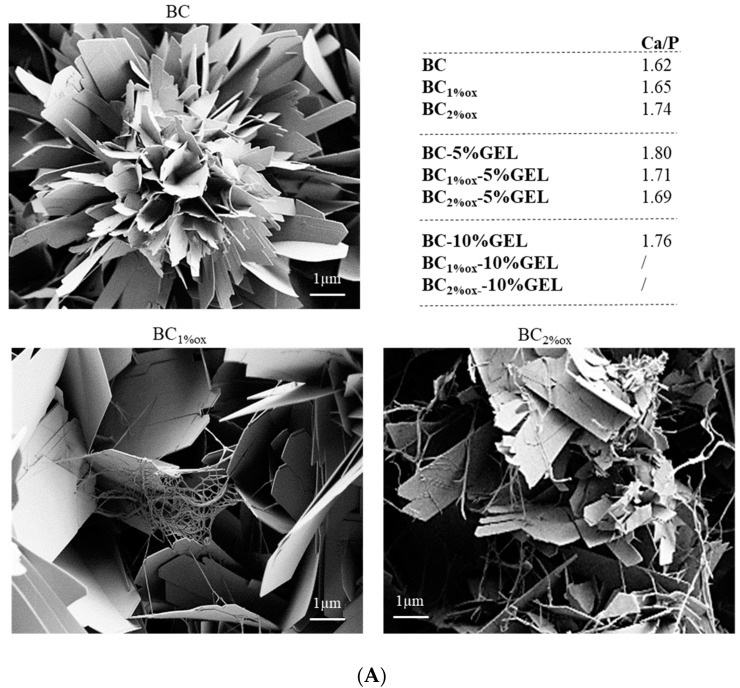

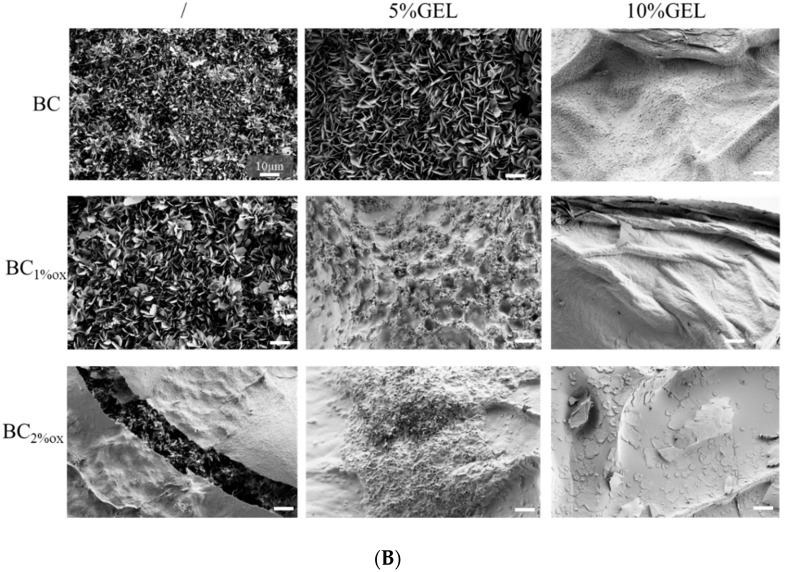
SEM micrographs of BC and BC–GEL membranes after 6 h of incubation in 10× simulated body fluid (SBF) media, containing non-oxidized (**A**); and oxidized (by 1% and 2% periodate) (**B**) BC. The table inset (**A**) identifies the Ca/P ratio extracted from the energy dispersive X-ray spectroscopy (EDX) data. The white scale bar presenting a 10-µm length is applicable to micrographs under (**B**).

SEM imaging of GEL-free, native, and BC1%ox (Figure 4A) demonstrated the presence of plate-like minerals with sharp edges and defined (flower) shapes, which in the case of oxidized BC were intertwined with dangling fibrils. The latter was more obvious in 2% oxidized BC, where smaller plates were formed. Such plate-like or platelet crystals growing perpendicular to the substrate surface were demonstrated within coatings on metallic surfaces, all having an octacalcium phosphate composition (OCP) [31]. On the other hand, the EDX-derived Ca-P ration between 1.62 and 1.65, being typical for coatings with nonstoichiometric HAp, are also discussed in the FTIR/XRD data analysis (in the following paragraphs). The presence of 5% GEL over the native BC supported the further growth of minerals with the same structural pattern, but with a higher Ca/P ration (1.80). In addition, there was a random presence of plate-like mineral shapes, which could have been related to non-uniform surfaces, as well as the presence of pores (Figure 2C,D). In membranes with 10% GEL, the EDX did not identify the presence of apatite phase. Indeed, there was the presence of a continuous white layer, most probably composed of carbonated mineral phase as either free carbonate or the substituted apatite (if a CO_3_^2−^ ion substitutes the OH group, type A is formed, while substitution of PO_4_^3−^ results in type B carbonated HAp [35]), as will be later demonstrated by FTIR. As the mimicking of host tissue is a major intention during organic–inorganic scaffold construction, the most suitable CaP mineral for biomedical applications presents low crystallinity and nonstoichiometric apatite [36]. Our data (Ca/P = 1.62–1.8) indicated the formation of such biomimetic, nonstoichiometric HAp [19,37].

**Figure 5:**
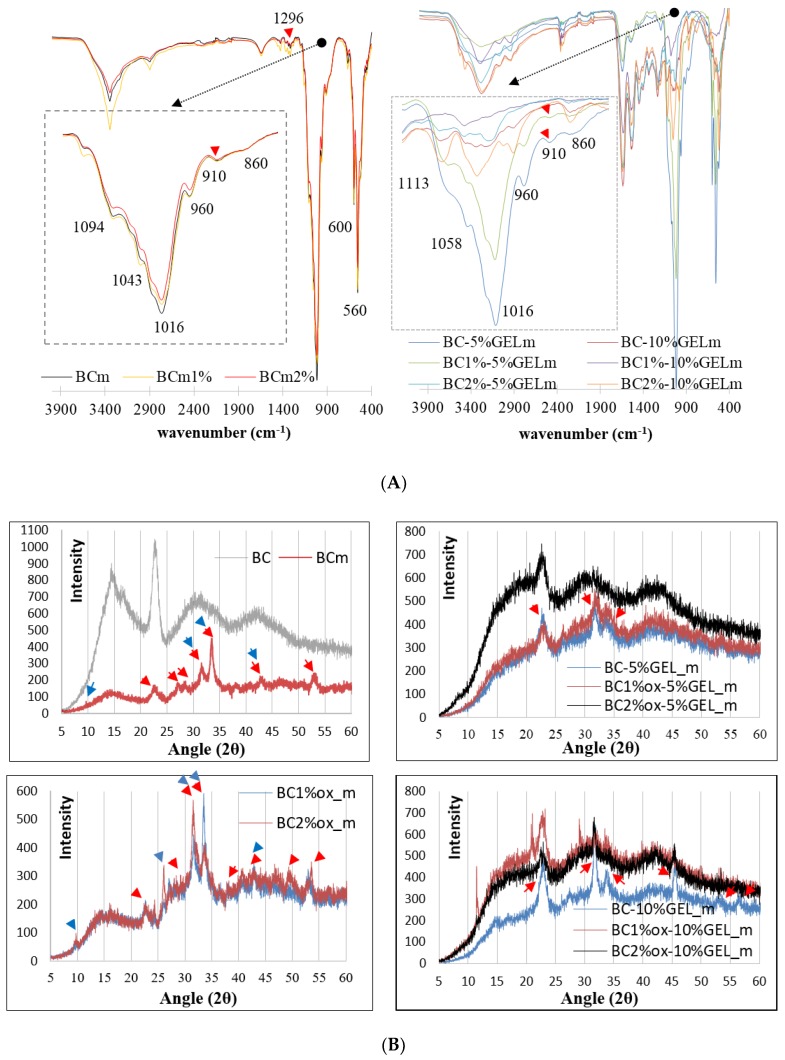
(**A**) FTIR and (**B**) X-Ray Diffraction (XRD) patterns of selected BC–GEL membranes with different periodate and GEL concentrations, after a 6-h incubation process in 10× SBF media at room temperature. Red and blue triangles are assigned to the HAp and OCP phases, respectively. The subscript m stands for mineralized samples.

The FTIR data (Figure 5A) aided us in identifying apatite-related bands, i.e., phosphate (asymmetrical stretching at 960, 1016, and 1094 cm^−1^, and bending at 560 and 600 cm^−1^), carbonate (1450 and 860 cm^−1^), and hydroxyl bands (bending at 3564 and 630 cm^−1^ [38]). CO_3_^2−^ ion presence at 860 cm^−1^ (in BC–GEL membranes) was already related to the substitution of phosphate ions within the apatite, resulting in B-type HAp [39]. On the other hand, the characteristic octacalcium phosphate (OCP) bands at 1296 and 910 cm^−1^ were related to P–OH stretching and O–H in plane banding of the HPO_4_^2−^ group, respectively. The presence of the PO_4_^3−^ doublet at 600 and 560 cm^−1^ in composites suggested that the precursor phase of HAp is OCP [40]. Interestingly, the latter bands were absent in samples with the highest GEL content, most probably due to a carbonate layer identified by SEM (and EDX). Combining data, the occurrence of a mixture of different Ca-P phases can be suggested, rather than a certain phase.

The XRD diffractograms (Figure 5B) presented mineralized membranes and native BC membranes as a control. The BC diffraction pattern demonstrated three main peaks, characteristic for cellulose I at 2θ = 14.2°, 16.6°, and 22.4°, assigned to the reflection planes (110), (110), and (200), respectively [10]. Mineralization of membranes resulted in several new (pointed arrows) peaks related to HAp (red arrow pointed peaks at 2θ = 26°, 32°, 39°, 49°, and 53°) and OCP (overlapping, blue arrow pointed peaks), indicating their coexistence in formed coatings. OCP has presented a hydrodynamically metastable phase, which may have hydrolyzed in a stable HAp phase [41]. The same study demonstrated that 6-h hydrolysis of OCP within a biomimetic coating resulted in the coexistence of integrated OCP/HAp minerals. In our experiment, the incubation was also terminated within 6 h, which we assume accommodated the time for formation and partial hydrolysis of OCP, the latter being explained by the absence of a peak portion at ~5° in our data, specific to the OCP phase. Further comprehensive analysis of parameters of crystal planes, which may distinguish between OCP and HAp phases, was not accomplished within the presented study.

On the other hand, a broad, amorphous peak in the 15–25° region was found in GEL-containing membranes, diminishing the previously described bands in line with an increase in GEL percentage and periodate concentration (i.e., oxidation extent). More pronounced bands were observed in BC–GEL membranes containing native BC, suggesting the surface chemistry and nano(micro)surface structuring of mineral formation. As evidenced by CFM, the periodate oxidation allowed more intimate GEL inclusion within BC, forming a denser structure with limited space for SBF ion diffusion and consequently limited, less effective precipitation. Comparing this with XRD references and recent review data [42], we suggest the possible presence of both amorphous calcium phosphate (ACP) forming at the initial incubation in SBF (as suggested by the EDX data) and OCP formed upon further incubation of ACP into saturated SBF (according to the characteristic, plate-like morphology seen by SEM). Both were hardly related to the EDX data, which we assume to have been an artifact of carbonated coverage with phosphate absence. While the additional washing may have partially resolved the problem, it was avoided since it was expected to alter the minerals’ state due to their metastable nature.

### 3.4. Physiological Data

Membrane swelling is an important parameter in GTR applications, as in clinical use it is placed in a defect site (e.g., a periodontal pocket). Apart from being a barrier for fibroblast infiltration within the bone part, it is desirable that the membrane act as a filler for the defect by delivering appropriate (morphological chemical, mechanical, etc.) cues for appropriate and efficient regeneration of bone tissue. Swelling of our hydrogel-like membranes is dependent on the polymeric network crosslinking density, the hydrophilicity of the polymers, and their concentration, explaining the highest values in the pure, OH-rich BC membrane. Indeed, the polyglycosidic chains of BC are surrounded by water molecules in the form of gel stabilized by H-bonding [43]. The inclusion of GEL reduces the relative swelling, especially in cases of periodate-treated BC, due to the introduction of GEL molecules within the BC (as suggested by CFM), forming a denser network with closer molecule contact. Further, the GEL included within BC is exposed to a freeze-thawing process during processing, which fosters the linking with BC (similar to PVA–BC composites [44]). The effect intensifies with GEL concentration, leaving less space for water molecules. The EDC–medicate linking even further arrests the GEL, preventing further swelling.

**Figure 6:**
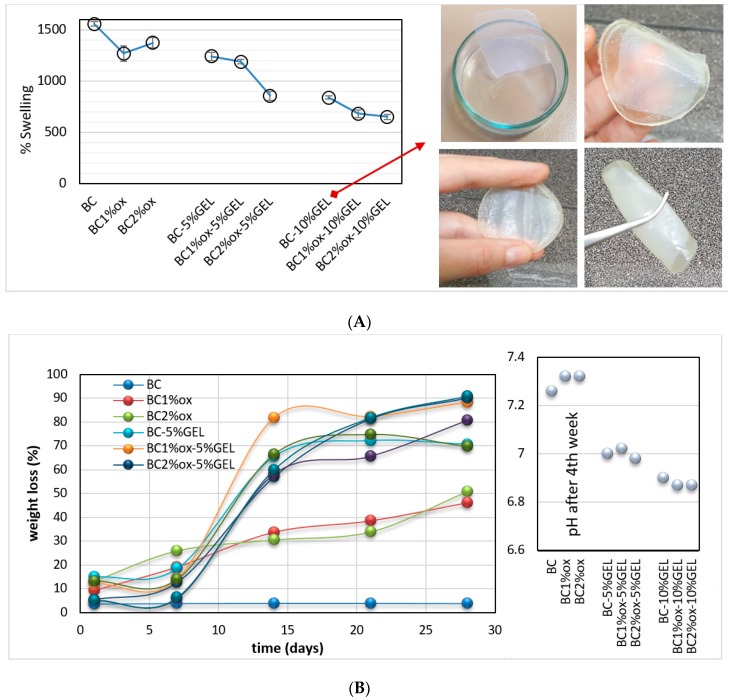
(**A**) Swelling % at equilibrium and (**B**) weight loss % after 1–28 days of incubation with scatter data of pH obtained from respective incubation solutions after the 28th day of incubation (b). Photographs were taken on dry and fully swollen BC–10%GEL membrane, with simplified demonstrations on its handling (stretch and bend). The results presented are mean values (*n* = 5; SD < 0.1 for weight loss and 0.01 for pH).

In clinics, GTR membranes should balance degradation and tissue regeneration rates [45]. Therefore, it should not be absorbed in the frame of 3–4 weeks, while it should completely degrade after completing the regeneration, so that surgical removal is avoided [46]. Simplified, enzyme-free SBF media were used in a degradation test due to high collagenase diversity and concentration inconsistency within a real scenario [47]. We observed progressive degradation within oxidized BC with up to 50% weight loss (in both 1% and 2% periodate-oxidized BC), significantly differing from < 4% degradation in native BC. The oxidized BC was prone to hydrolysis due to reduced crystallinity (as demonstrated by FTIR data), with simultaneous introduction of aldehyde groups promoting the degradation. Data evidence trend in BC degradation profile, meaning that even heterogeneously, oxidation could be controlled to the extent that implant duration met the kinetics of physiological regeneration. In GEL-containing membranes, the degradation was less linear, continued, and was most prominent between the first and second week. We assume that such a profile was a consequence of GEL degradation, postponed by EDC chemistry, which was also demonstrated by a slight lowering of the pH (Figure 6B) in degradation media of membranes with the highest GEL content and the highest, up to 90%, weight loss in a 4-week period.

### 3.5. Cell Testing

**Figure 7:**
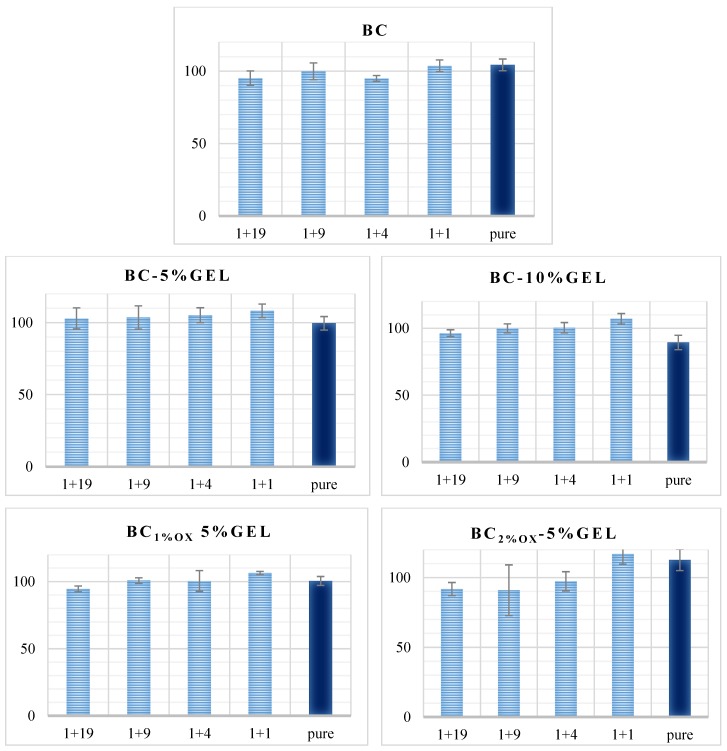
Dehydrogenase activity of MRC-5 cells exposed to eluates of the sample for 72 h. The viability of cells treated with medium was set as 100% (*y* axis). Eluate (pure) and different dilutions are presented on the *x* axis.

The cytotoxicity of BC and BC–GEL membranes toward MRC-5 cells was tested in two ways, i.e., indirectly by exposure to eluted products in a 72-h period and directly by cell exposure to the membrane surface. The pure eluates from tested membranes did not decrease the dehydrogenase activity of MRC-5 cells (Figure 7) in the elution test, which was direct evidence for noncytotoxicity of the eluted molecules (nonlinked, short-chain gelatin protein, oxidized cellulose segments, hydrolyzed crosslinking agent), which may have been potentially released from the material within the 72-h incubation period.

**Figure 8:**
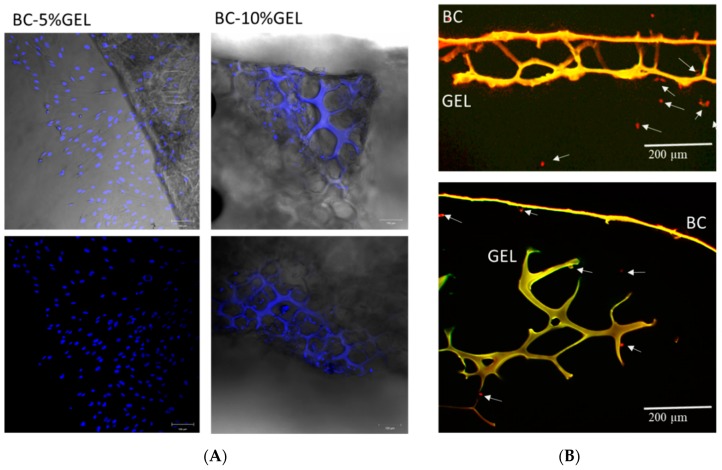
(**A**) CFM images of BC–5%GEL and BC–10%GEL membranes on whole mounts. Cell nuclei as well as autofluorescence of the samples are seen (blue); (**B**) CFM images of membrane cross-sections with assigned BC and GEL segments and pointed-arrow cells. Cells were labeled red during image processing for better contrast and visualization.

In direct testing, cell behavior was observed on the membranes. Cells were scattered over the samples with a higher density at the edges of the material (Figure 8A) due to migration from the densely cell-populated bottom of the cell culture plate. An image taken from the edge of the membrane BC1%ox–5%GEL (Figure 8A) appears to show that cells migrated from the BC side to the bottom (GEL side) of the well due to affinity with this material. The cell collection from the denser (BC) side, similar in all investigated membranes, showed that almost no cells attached.

The BC surface of the membrane was fully covered by gelatin, and it is known that extracellular matrix proteins generally increase cell adhesion [48], and aldehyde BC supports cell adherence and growth [23]: In our case, improved adhesion and proliferation were not observed. We speculate that GEL conformation was a major factor for such an effect. Indeed, not only the presence of GEL itself, but also the right conformation exposing the cell adhesion units were of utmost importance for cell adhesion. A recent work [49] demonstrated that chemical identity change, as well as conformation and availability of free binding motifs (such as GxOGER and RGD) resulting from the addition of GEL to collagen and finally cross-linking, have a profound effect on the ability of cells to adhere to these formulations. In the helical form, RGD motifs are constrained and as such cannot bind the integrin from the cell, while in an unfolded (random) state RGD-containing strands are more flexible and free to coordinate with Mg ions, bonding to cell integrins. As was said, the low attachment on the BC side (with a thin, layer-like GEL covering) could be a consequence of high consumption of carboxylate groups of glutamate and aspartate during the EDC cross-linking chemistry, which are crucial for binding to integrins on the cell surface. The same research offered evidence that EDC crosslinking ablates integrin-dependent cell activity on both two-dimensional and three-dimensional architectures, while a three-dimensional scaffold structure also leads to a high level of nonspecific interactions remaining on three-dimensional samples even after a rigorous washing regime [49].

A particular part of the cell analysis was devoted to the examination of membranes as barriers to cells. During conventional periodontal treatments, the void left by damage in the periodontal complex tissue is normally populated by epithelial cells rather than periodontal cell types due to 10-times faster migration [50], so the idea behind GTR barrier membranes is to stop their proliferation while securing space for periodontal cell types. The results were collected from direct exposure, where cells were tested for their ability to proliferate through the BC part of the membranes, examining the membrane cross-sections. In all cases, no proliferation was observed through the membrane (Figure 8B). As we already stated, the BC itself, or intertwined with GEL, presented a dense network, with pore sizes far below the (fibroblast) cell size. Very few cells were seen proliferating from the bottom, porous part (Figure 8B), where barrier function was not expected. This was a very positive clue that such a porous GEL environment was attracting cells, and future tests with osteoblasts will more accurately identify if the porous part acts as a stimulating environment for enhanced osteogenesis within the alveolar bone part destroyed by periodontal disease.

## 4. Conclusions

In summary, we demonstrated a facile (freeze/thawing–crosslinking) procedure for the development of biobased, composite membranes from (oxidized) BC and highly microstructured GEL, thus confirming our first hypothesis. Particular focus was given to a GEL microstructuring assessment on one side and in situ mineralization on another, the first as a prerequisite for judging suitability for cell infiltration within the porous part, and the latter as an approach to introducing the biologically relevant mineral phase (OCP and HAp) in composite membranes. In line with these, the second hypothesis was only partially confirmed, since BC–GEL membranes with higher GEL concentrations and BC oxidation levels were hardly resolved in term of mineralization assessment, due to lack of P tracks (with relevant EDX data). We assume this to have been an effect of denser structures with limited space for SBF ion diffusion in membranes with more intimate GEL inclusion within BC. Our third hypothesis was also confirmed, as MRC-5 cells were found to attach well on GEL porous sites, with no attachment on the BC (GEL coated) side, which we speculate to have been an ablation effect of dominant β-sheet conformation and crosslinking in GEL that consumed the attaching moieties. The physiological (high swelling, maintainability, degradation, and retained pH) performance and positive, secured noncytotoxicity and barrier toward fibroblast proliferation with supported attachment on GEL sites were found to be promising attributes for GTR applications.

Further cell study, including an assessment of products metabolized by seeded osteoblasts, is expected to precisely elucidate the porosity impact on the regeneration process, as well as to give a qualitative measure on the advancement of the proposed material over the collagen membrane as a GTR benchmark product.

## Supplementary Materials

The following are available online at https://www.mdpi.com/2079-4991/9/2/303/s1, Figure S1: Confocal fluorescence microscopy images of BC–GEL membranes, without and with 5% and 10% GEL, and (non-, 1%, and 2%) oxidized BC. Top and bottom positions are relative to freezing plate position at freezing stage.
