# Supplementary material for: Microstructured and Degradable Bacterial Cellulose–Gelatin Composite Membranes: Mineralization Aspects and Biomedical Relevance
Source: Nanomaterials (Basel). 2019 Feb 22;9(2):303. doi: 10.3390/nano9020303 (PMC6409525; doi:10.3390/nano9020303)
Supplement: Supplementary file 1 [file nanomaterials-09-00303-s001.pdf]

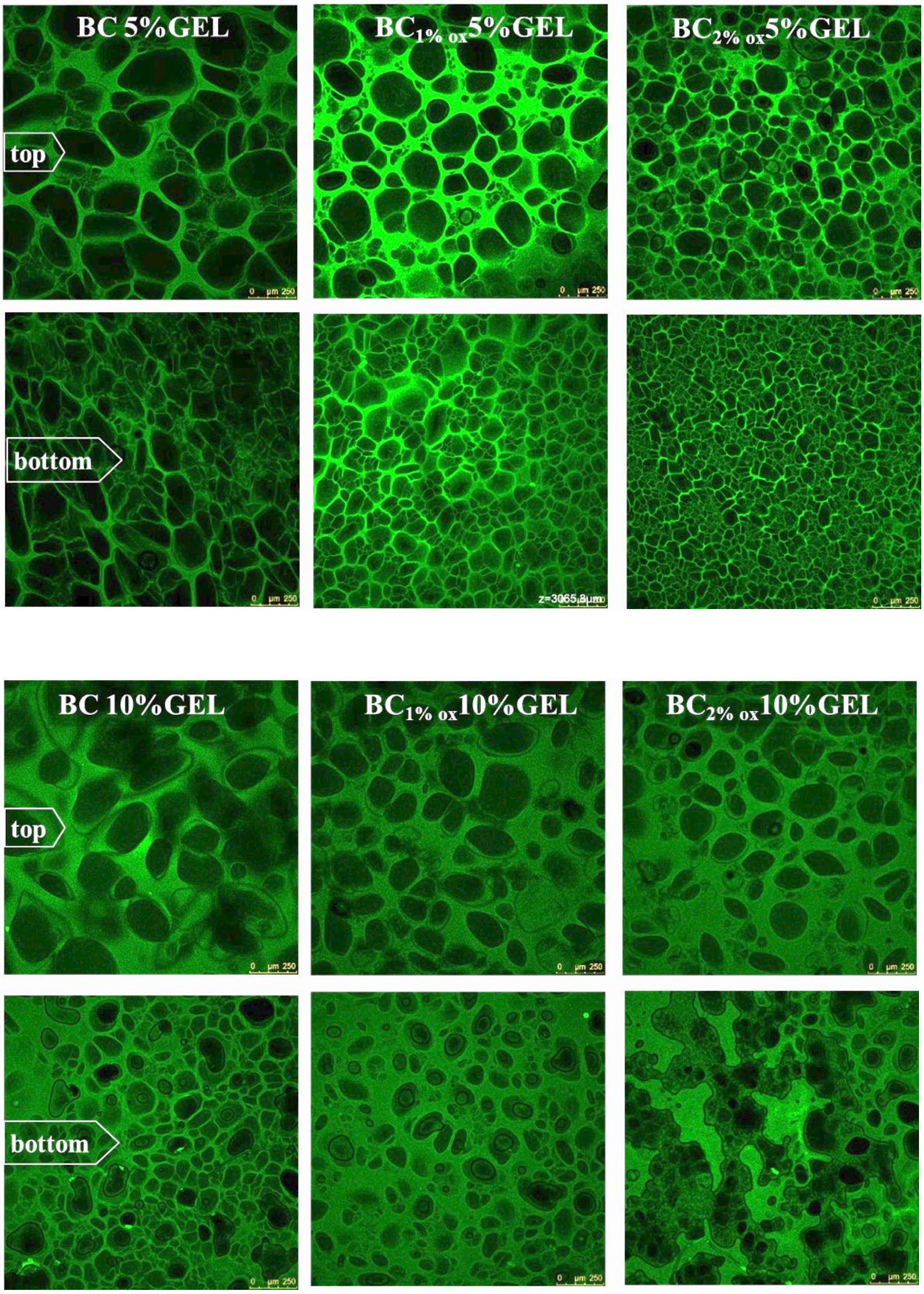

Figure S1. Confocal fluorescence microscopy images of BC–GEL membranes, without and with 5% and 10% GEL, and (non-, 1%, and 2%) oxidized BC. Top and bottom positions are relative to freezing plate position at freezing stage.
